# Online Reverse Supply Chain: New Layout to Promote Recycling Industry in China, 2015–2019

**Published:** 2020-01

**Authors:** Di WU, Juhong CHEN, Ruijun ZHANG

**Affiliations:** School of Economics and Management, Xi’an University of Technology, Xi’an, China

## Dear Editor-in-Chief

As the saying goes: one man’s trash is another man’s treasure (1).The development of Internet technology has brought earth-shaking changes to the recycling industry, due to the “Internet recycling” policy proposed by the Chinese government since 2015. In 2017 alone, Loving Recycling, a recycling company in China, recycled more than 11 million WEEEs (Waste of Electric and Electronic Equipment) through Internet channels. Online recycler actively develops online reverse supply chain based on website, app and reverse vending machines, which can not only increase the recycling amount, but also promote recycling rate and sustainable utilization of resources (2). However, this does not mean that its development is smooth sailing. As a new type of recycling mode that has been in existence for less than five years, we must clearly identify the challenges it faces and propose targeted solutions.

In this letter, we want to emphasize several important issues that online recyclers need to consider in their transactions with traditional offline third-party recyclers (TPR) and consumers. We believe that the attention and solution to these problems can not only improve the profits of online recycler and optimize the recycling mode, but also promote the consumers’ awareness of environmental protection. After understanding such issues, scholars can participate in contributing their own views and research to further promote the development of this field.

### Optimizing recovery services

The biggest advantage of online recycling lies in its services in terms of safety and convenience. This is due to the mobility of TPR, which makes it difficult to do home recycling. In addition, after TPR recycles WEEEs, it is difficult for consumers to get in touch with it and to know the condition of WEEEs. This has also led to frequent personal phone privacy leaks in recent years. However, online recycling does not have the above problems due to home recovery, logistics tracking and professional data erasure technology. For example, Loving Recycling entered a partnership with Blancco, the world’s top data security company, in 2017 to improve the technical level of permanently erasing information in electronic products. Although the high service level can effectively improve the recovery volume, it will also lead to an exponential increase in the service cost. In addition, online recycler also needs to adopt different service configurations for different kinds of WEEEs. For example, consumers trying to recycle TV sets and mobile phones have different preferences for services.

### Promoting consumer’s preferences

The change of consumers’ preferences for online and offline channels directly affects the amount of online recycling. Despite the high volume of online recycling transactions, the market as a whole is still monopolized by offline recycling.

On the one hand, this is because consumers use offline recycling for many years and it is difficult to change their habits. On the other hand, in some areas where the popularity of the Internet is not high, online recycling is rarely known. For example, in some big cities such as Beijing and Shanghai, the proportion of consumers using online recycling is significantly higher than that in some cities in northwest China. Therefore, online recycler can actively publicize the advantages of online recycling in environmental protection, resource conservation and recycling services while raising prices, and strive to improve consumer’s preferences (3).

### Reducing conflicts with offline enterprises

As a new competitor, the high recovery price and service of online recycler will continue to share the market of offline channels and certainly cause dissatisfaction with TPR. For online recycler, failure to properly handle the relationship with TPR and maintain supply chain coordination may lead to TPR’s conflict behaviors such as opportunism and fraud, and damage the interests of all industry participants including TPR, consumers and itself. To avoid this problem, online recycler should incorporate TPR’s fair preference behavior into decision-making factors when pricing WEEEs (4). Furthermore, the multi-faceted communication and cooperation with TPR will also become the choice of online recycler to ease channel conflicts.

We believe that the development of Internet technology will prompt online reverse supply chain to gradually replace traditional mode as the mainstream of recycling industry in the future. On one hand, its price is bound to be higher than the price set by TPR, which will lead to the enthusiasm of consumers. On the other hand, online recycler’s more professional disassembly technology can not only reserve more resources, but also reduce harmful emissions to the environment. This will not only promote the publicity for environmental protection, but also be supported by government policies (5). For example, the Chinese government has formulated policies to give huge subsidies to online recycler every year.

This article highlights the concept, advantages, importance and several aspects needing promotion of online reverse supply chain. In order to accelerate the development of online reverse supply chain and further optimize the structure of recycling industry, we suggest this as a topic for future research and invite scholars, entrepreneurs, government agencies and other stakeholders around the world to share and discuss their views. These studies will not only help online recyclers and consumers, but will benefit the earth and all mankind.
